# Supplementary material for: Oxygen nanoclustering evades inverse Hall-Petch softening
Source: Nat Commun. 2025 Nov 27;16:10602. doi: 10.1038/s41467-025-66181-1 (PMC12660734; doi:10.1038/s41467-025-66181-1)
Supplement: Supplementary file 1 — Supplementary Information [file 41467_2025_66181_MOESM1_ESM.pdf]

Supplementary Information for

Oxygen nanoclustering evades inverse Hall-Petch softening

Xiaolong Yu[1,2], Xilei Bian[1,2*], Chang Liu[3*], Qing Wang[1,2*], Daniel Şopu[4], Daniel Kiener[5], Yifeng Li[6], Ge Wu[3], Yuan Wu[7], Yong Yang[8,9], Jürgen Eckert[4,5], Gang Wang[1,2*]

[1]State Key Laboratory of Materials for Advanced Nuclear Energy, Shanghai University, Shanghai 200444, China

[2]Zhejiang Institute of Advanced Materials, Shanghai University, Jiashan 314100, China

[3]State Key Laboratory for Mechanical Behavior of Materials, Xi'an Jiaotong University, Xi'an 710049, China

[4]Erich Schmid Institute of Materials Science, Austrian Academy of Sciences, Jahnstraße 12, Leoben 8700, Austria

[5]Department Materials Science, Montanuniversität Leoben, Jahnstraße 12, Leoben 8700, Austria

[6]Laboratory for Microstructures, Shanghai University, Shanghai 200444, China

[7]State Key Laboratory for Advanced Metals and Materials, University of Science and Technology Beijing, Beijing100083, China

[8]Department of Mechanical Engineering, College of Engineering, City University of Hong Kong, Hong Kong 999077, China

[9]Department of Materials Science and Engineering, College of Engineering, City University of Hong Kong, Hong Kong 999077, China

*Corresponding authors:
bianxilei@shu.edu.cn (X.L. Bian)
chang.liu@xjtu.edu.cn (C. Liu)
qingwang@shu.edu.cn (Q. Wang)
g.wang@shu.edu.cn (G. Wang)

This PDF file contains:

Figs. S1-S18
Tables S1-S5
References

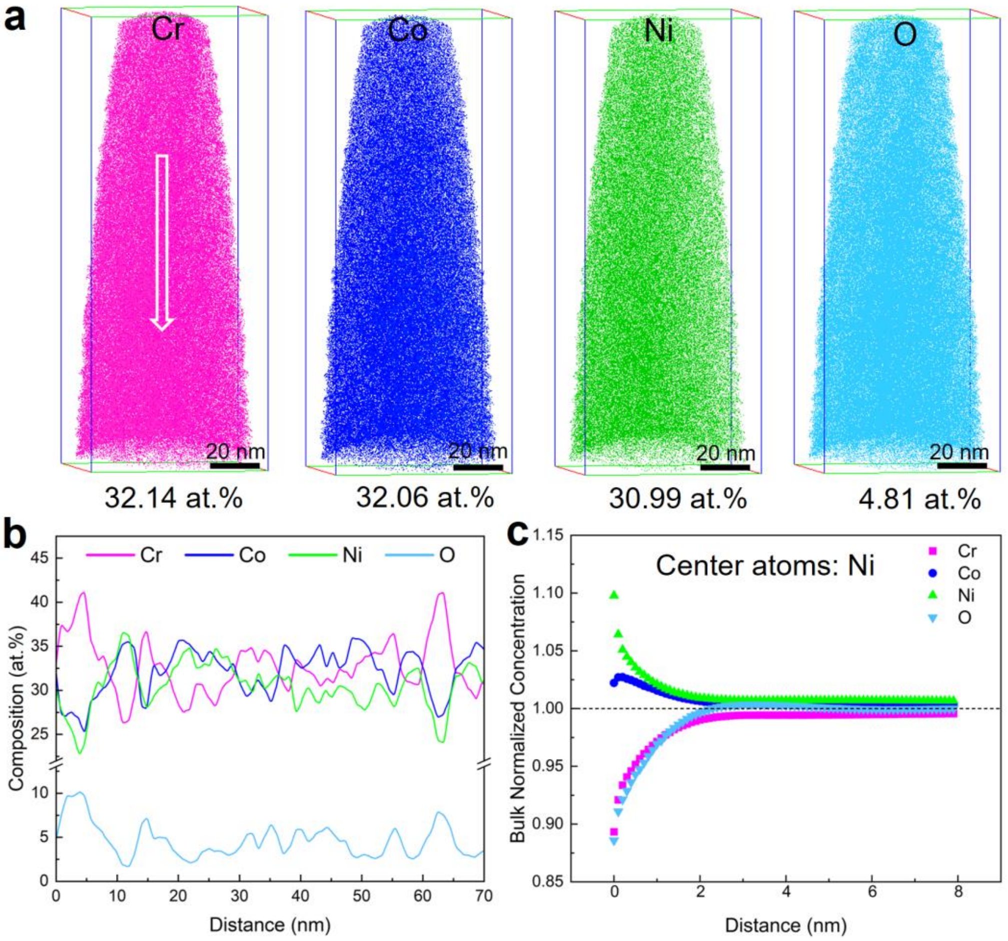

Supplementary Fig. 1| APT analysis of $(CoCrNi)_{95}O_5$ (O-5) MPEA. a Individual 3D elemental maps of as-deposited O-5 MPEA with chemical composition in atomic percent (at.%) based on APT analysis. **b** 1D compositional profile along the length direction of the arrow displayed in **a. c** The calculated RDFs from the APT data with Ni as the center atoms, showing pronounced chemical inhomogeneities. Note that the RDFs are normalized by the averaged composition for the APT-analyzed volume. Source data are provided as a Source Data file.

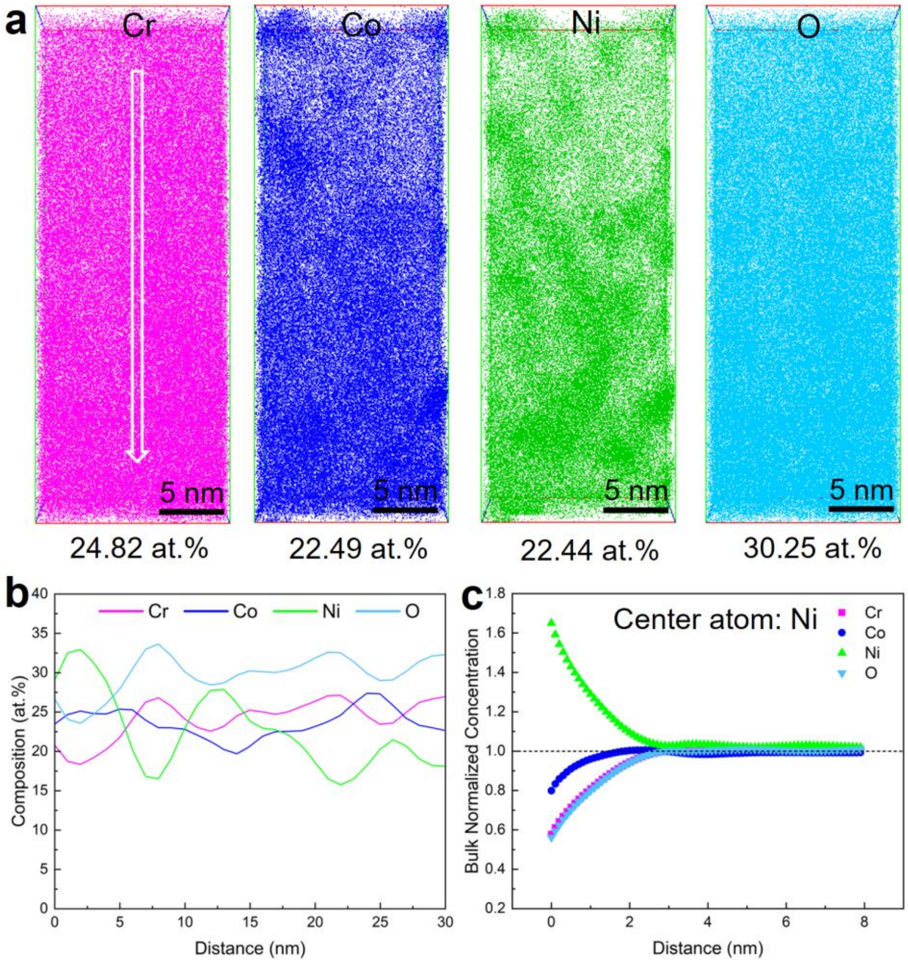

Supplementary Fig. 2| APT analysis of $(CoCrNi)_{70}O_{30}$ (O-30) MPEA. a Individual 3D elemental maps of as-deposited O-30 MPEA with chemical composition in atomic percent (at.%) based on APT analysis. **b** 1D compositional profile along the length direction of the arrow displayed in **a. c** The calculated RDFs from the APT data with Ni as the center atoms, showing pronounced chemical inhomogeneities. Note that the RDFs are normalized by the averaged composition for the APT-analyzed volume. Source data are provided as a Source Data file.

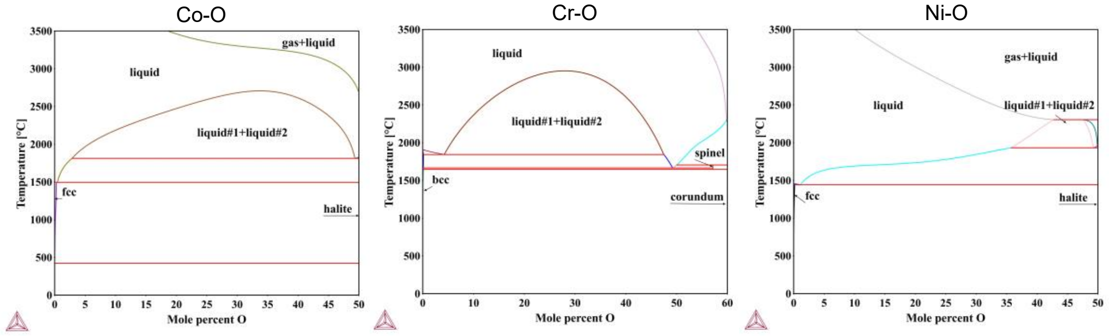

Supplementary Fig. 3| Binary Co-O, Cr-O, and Ni-O phase diagrams based on Thermo-Calc TCOX10 database[1]. In all three metallic elements, the solubility of O is below 5 at.%.

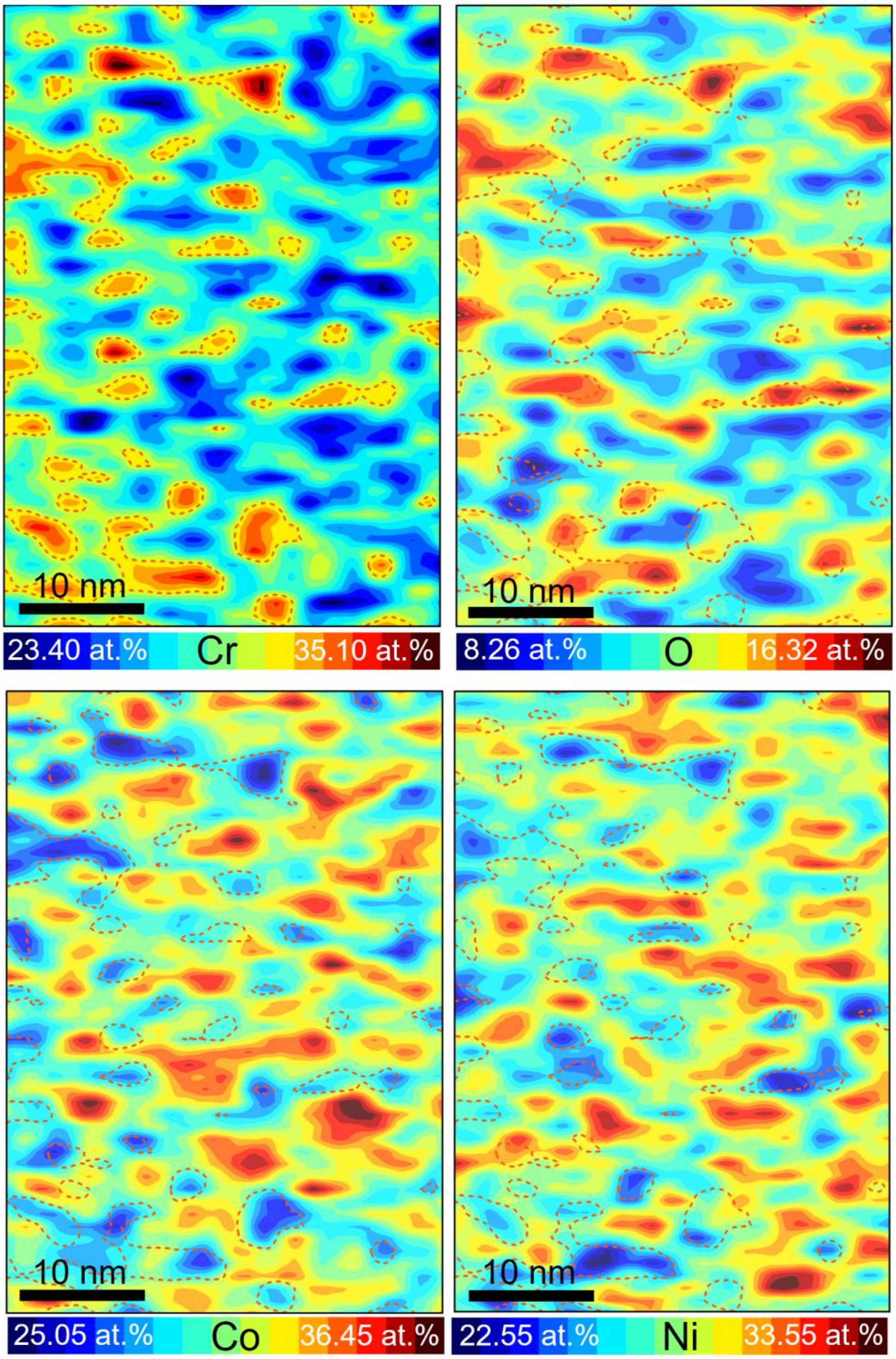

Supplementary Fig. 4| Chemical inhomogeneity on the 2D element concentration projection plane of O-13 MPEA. This is the plane-view projection of a 1 nm thick slice derived from the 3D-APT reconstruction map. The dotted orange lines on each plot correspond to the iso-concentration surface of 30.50 at.% Cr. Source data are provided as a Source Data file.

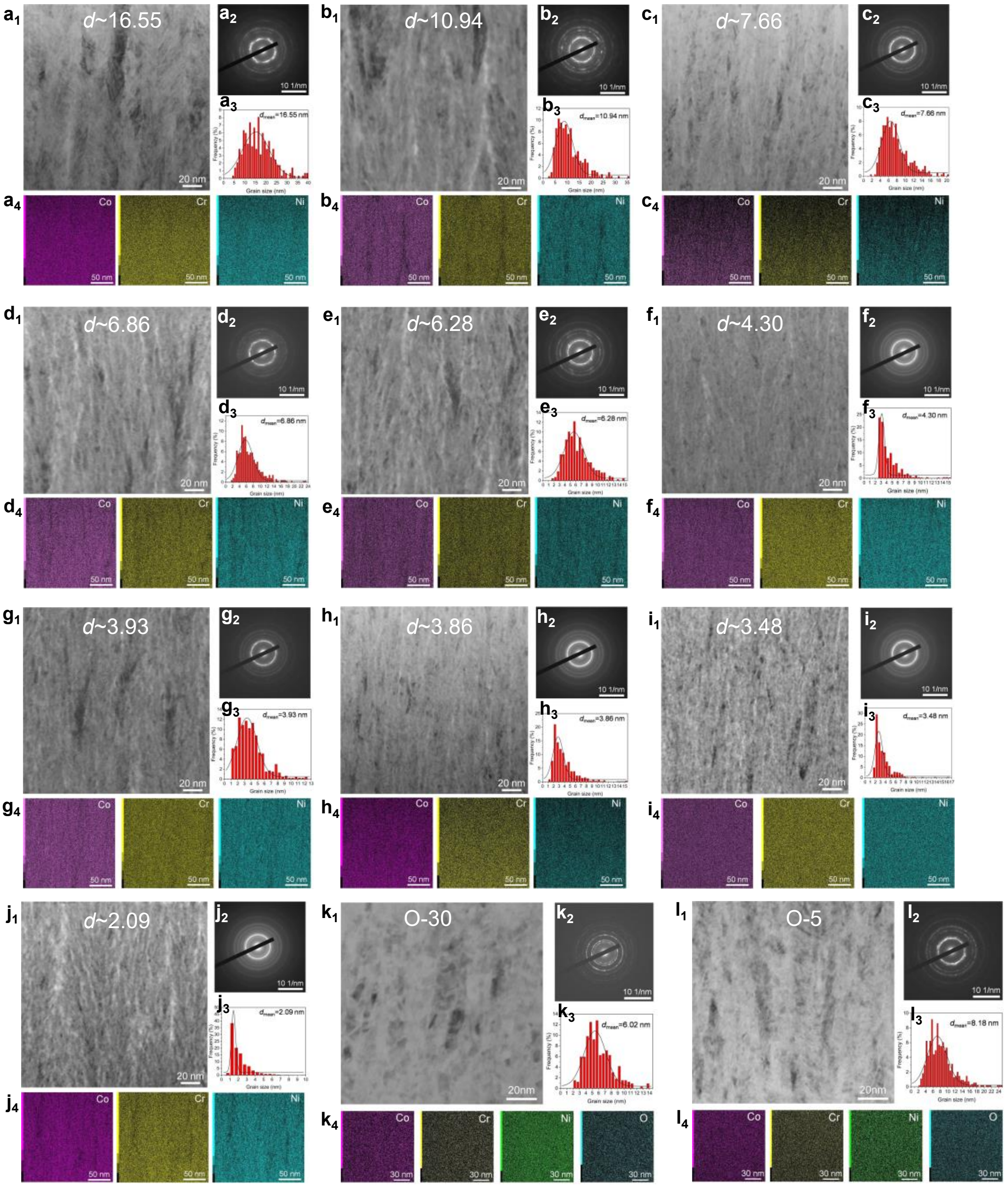

Supplementary Fig. 5| Microstructure of pure CoCrNi (labeled as *d*~ grain size), O-30, and O-5 MPEAs. a1-l1 Typical BF-STEM images. **a2-l2** The corresponding SAED patterns. **a3-l3** Diameter distribution of the columnar nanograins. **a4-l4** The corresponding EDS maps of the same BF-STEM images. Source data are provided as a Source Data file.

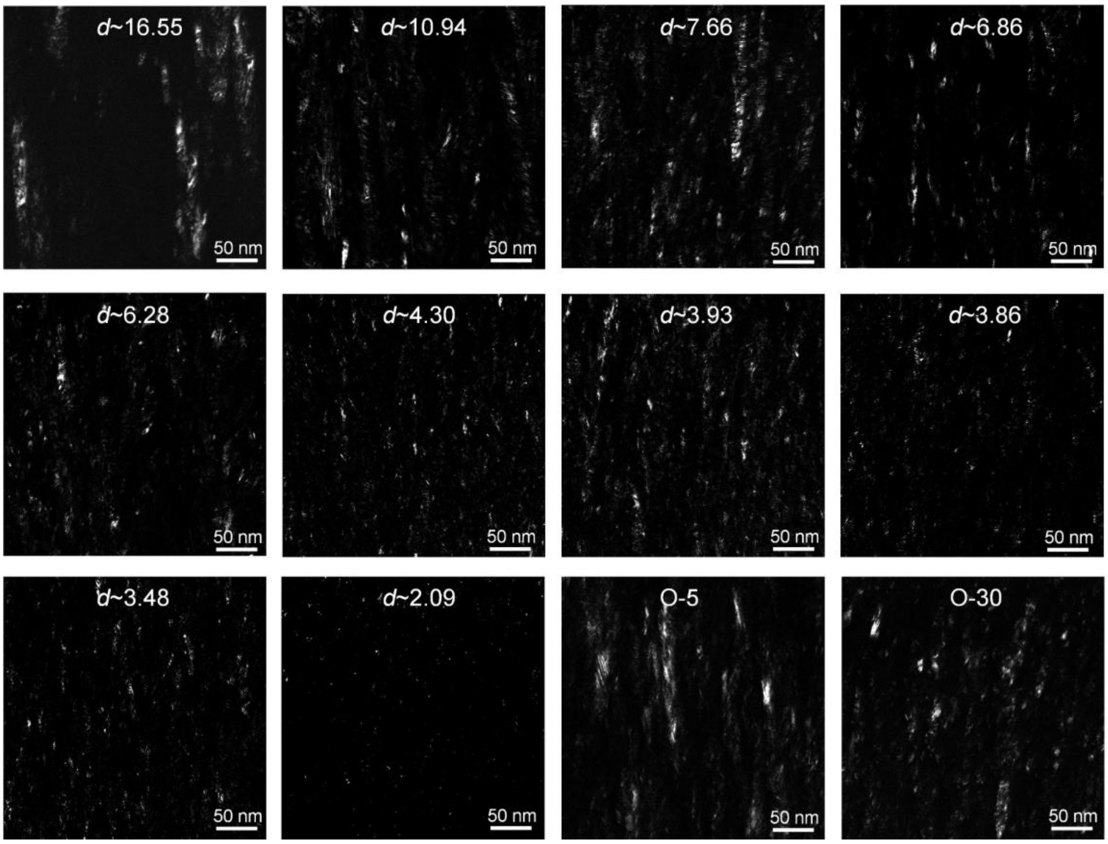

Supplementary Fig. 6| DF-TEM images of pure CoCrNi, O-5, and O-30 MPEAs.

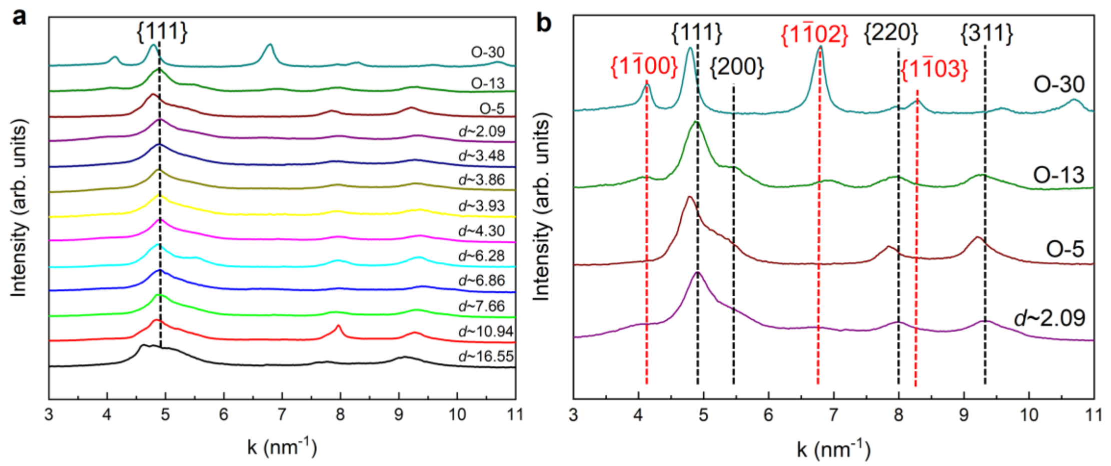

Supplementary Fig. 7| Diffraction profiles deduced from SAED patterns of pure CoCrNi, O-5, O-13, and O-30 MPEAs using PASAD-tools[2]. a Diffraction profiles of all samples, with {111} peak intensities normalized to the *d*~6.86 sample. The average {111} interplanar spacing of CoCrNi was 2.04 Å. **b** Enlarged profiles of O-5, O-13, O-30 and *d*~2.09 samples, showing the lattice expansion induced by oxygen doping. Source data are provided as a Source Data file.

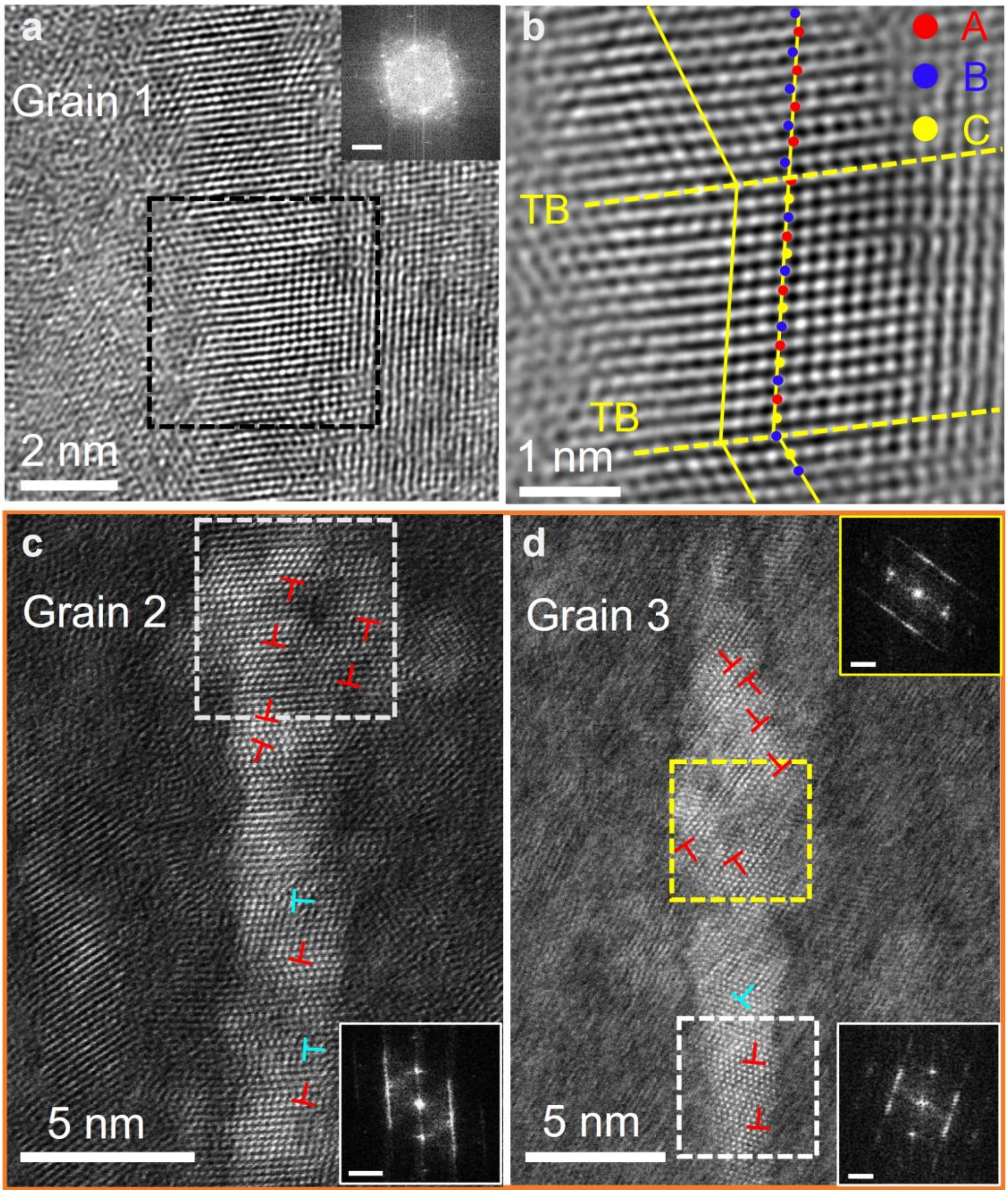

Supplementary Fig. 8| TEM characterization of the ultrafine columnar nanocrystals in O-13 MPEA. a BF-HRTEM image of a columnar nanograin with HCP phase. The inset depicts the corresponding FFT image. **b** Magnified BF-HRTEM image corresponding to the square area in **a**, demonstrating twin boundaries (TBs) as well as HCP phase arranged in ABAB stacking order. **c** and **d** Typical columnar nano-grains with different orientations containing a high density of partial dislocations, as verified by their corresponding FFT patterns (insets). Scale bar of insert = 5 1/nm. The red and blue "⊥" represent Shockley and Frank partial dislocation, respectively.

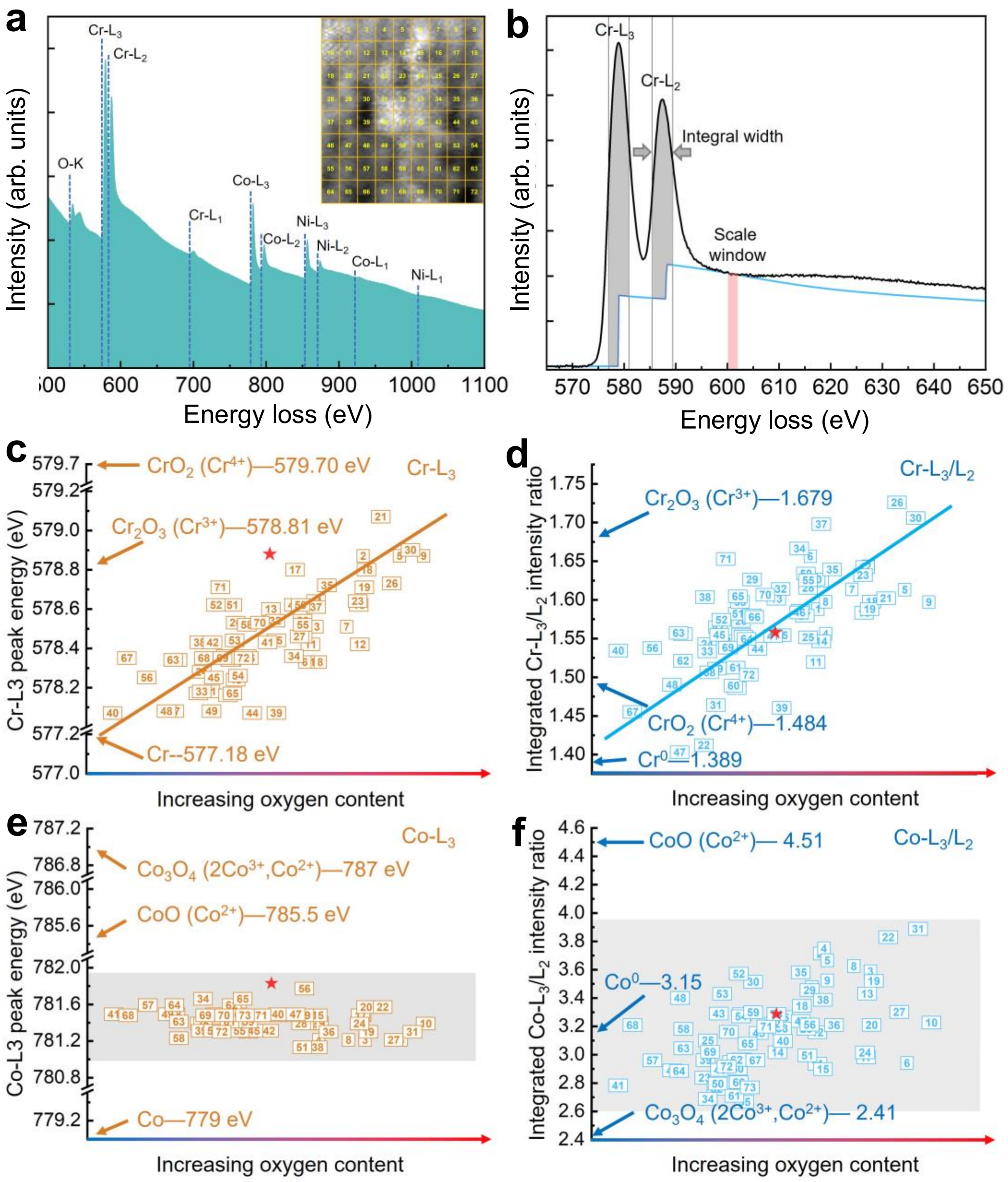

Supplementary Fig. 9| EELS analysis of the as-deposited material. a Full EELS spectrum of the O-13 MPEA. The inset shows the partition method used, i.e., the image is divided into 72 (8 × 9) subregions. **b** Spectral windows used to calculate the white-line ratio for a typical Cr-$L_{2,3}$ edge. Cr-L_3 (**c**) and Co-L_3 (**e**) peak energy dependence on oxygen concentration, whereby the label of the points indicates the regions of EELS collection. The symbol "★" corresponds to the average oxygen content of the whole plot. Integrated Cr-L_3/L_2 (**d**) and Co-L_3/L_2 (**f**) intensity ratio dependence on oxygen concentration. Source data are provided as a Source Data file.

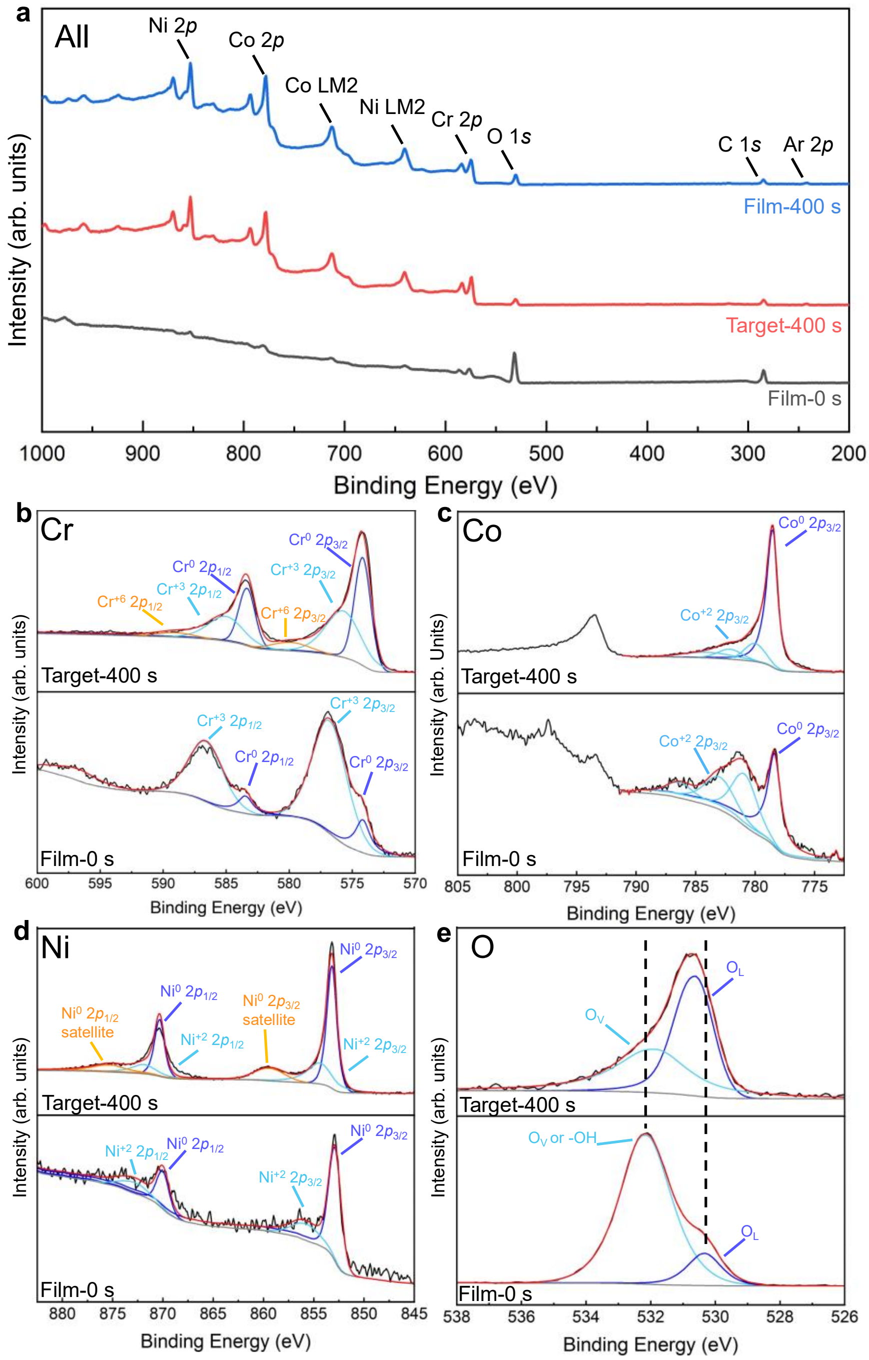

Supplementary Fig. 10| XPS spectra. a Overview XPS spectra of the O-13 MPEA for 0 s and 400 s etching time, and the pre-alloy target after 400 s etching time. **b-e** High-resolution XPS spectra for Cr, Co, Ni, and O, with deconvoluted peaks labeled with their corresponding chemical states. The O_L, O_V and -OH in (**e**) represent lattice oxygen, oxygen vacancy and the O species of the hydroxyl group, respectively. The black, gray and red lines represent the raw spectrum, background, and the overall fitting curve, respectively. Source data are provided as a Source Data file.

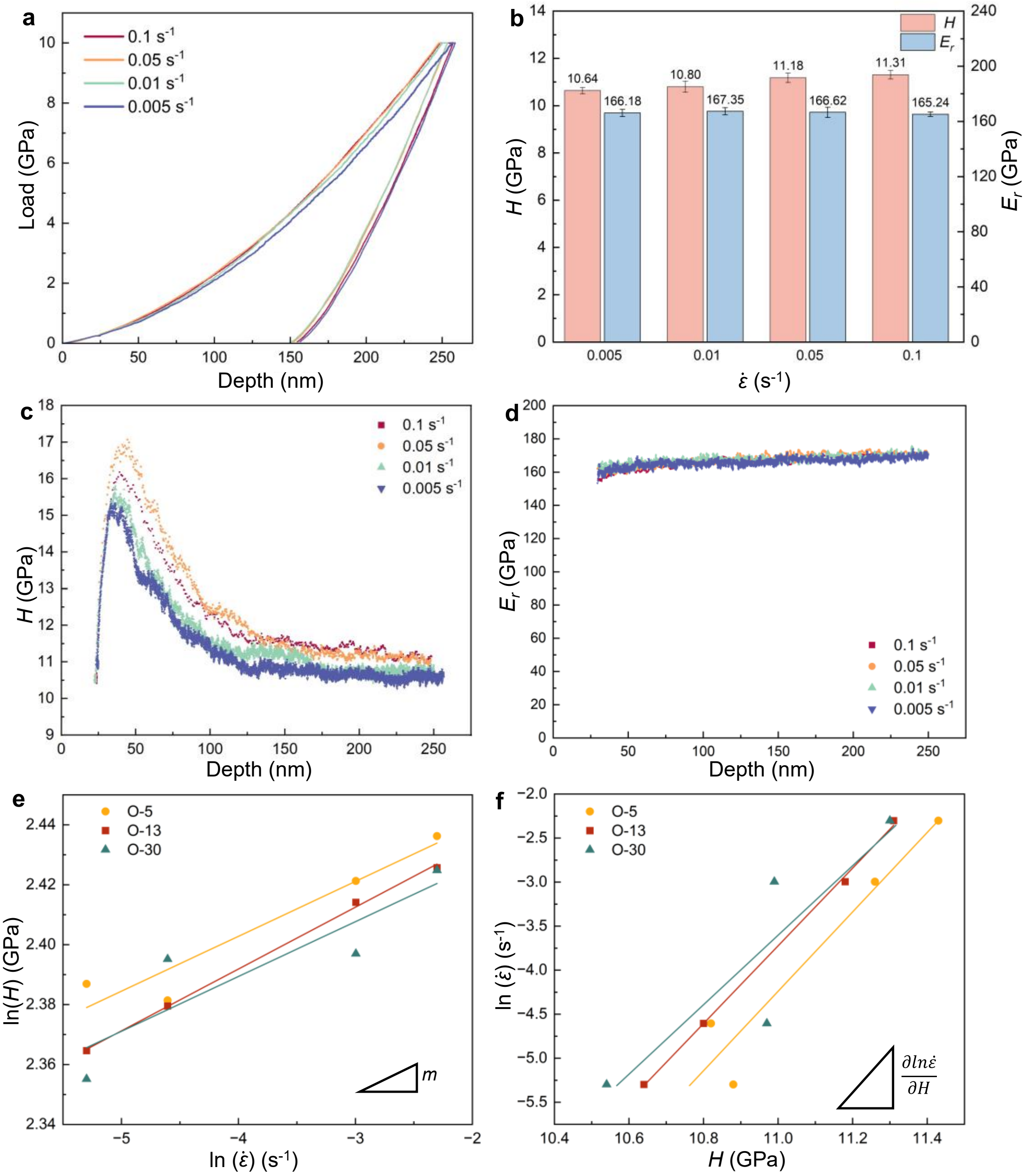

Supplementary Fig. 11| Nanoindentation results of the as-deposited O-13 MPEA tested with strain rates ranging from 0.005 to 0.1 s^{-1}. a Representative load-depth curves. **b** Statistical histograms of hardness (H) and reduced modulus (E_r). Error bars represent standard deviation. Variations of hardness and reduced modulus as a function of indentation depth are shown in **c** and **d**, respectively. **e** Double logarithmic curves of the hardness as a function of indentation strain rate. **f** Ln($\dot{\varepsilon}$) as a function of indentation hardness. Source data are provided as a Source Data file.

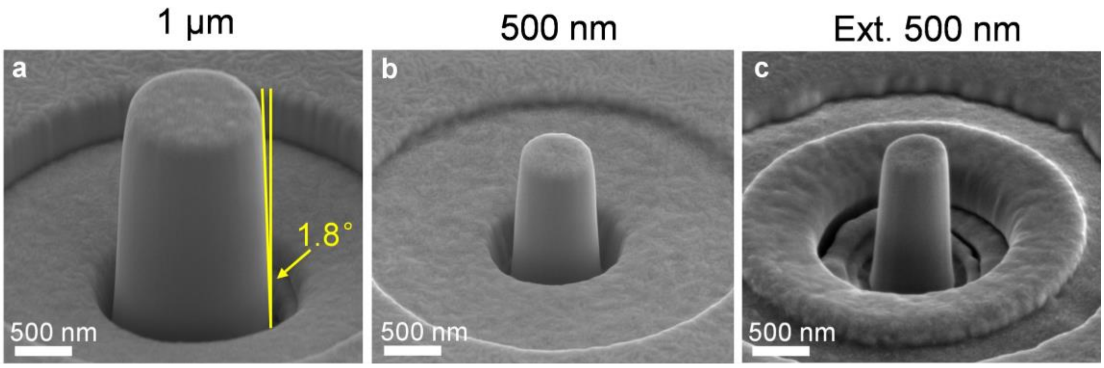

Supplementary Fig. 12| Morphology of as-fabricated O-13 micropillars. SEM images of as-fabricated micropillars with different diameters. **a** 1 μm. **b** 500 nm. **c** Ext. 500 nm. The taper angle of the micropillars is ~1.8°.

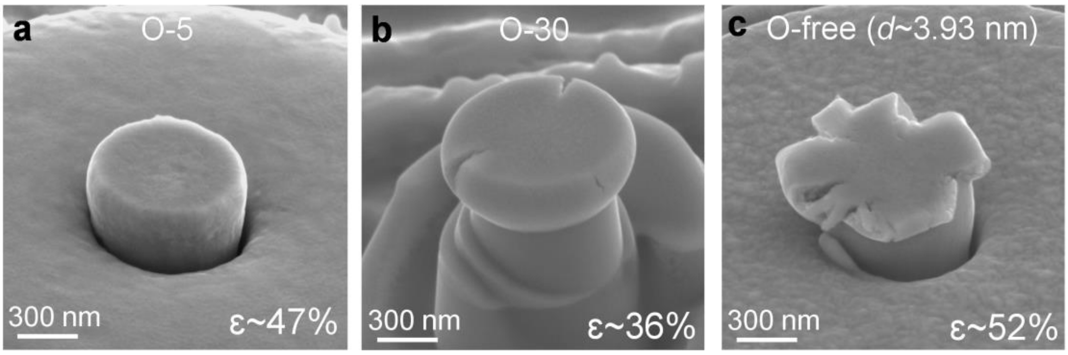

Supplementary Fig. 13| Morphology of the deformed micropillars. SEM images of **a** O-5 alloy, **b** O-30, and **c** O-free (*d*~3.93 nm) micropillars after deformation.

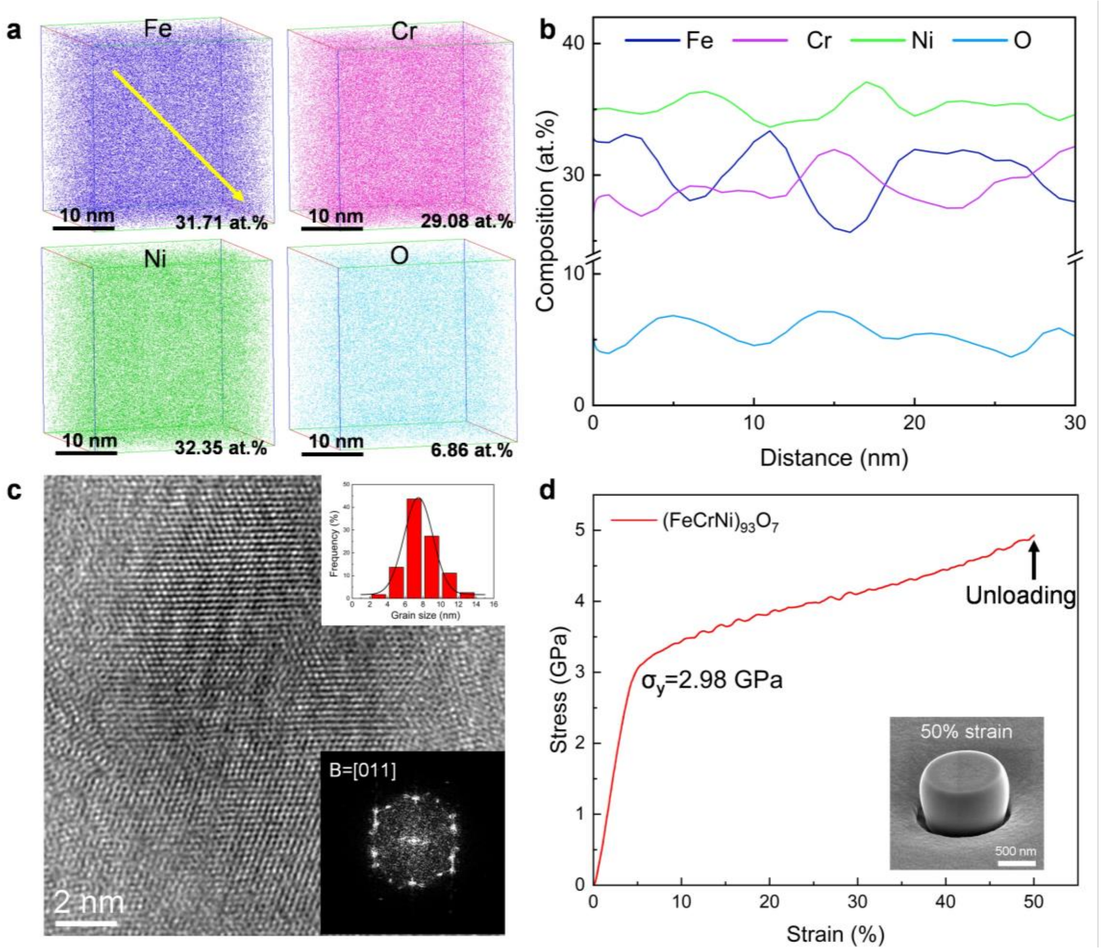

Supplementary Fig. 14| Composition, microstructure and mechanical properties of $(FeCrNi)_{93}O_7$ MPEA. a Individual 3D elemental maps comprised with specific element content. **b** 1D compositional profile along the arrow displayed in a. **c** HRTEM image along with the grain size distribution and corresponding FFT image. **d** Typical compressive stress-strain curve and corresponding SEM image of deformed micropillar. The diameter of the as-fabricated micropillars is ~700 nm. Source data are provided as a Source Data file.

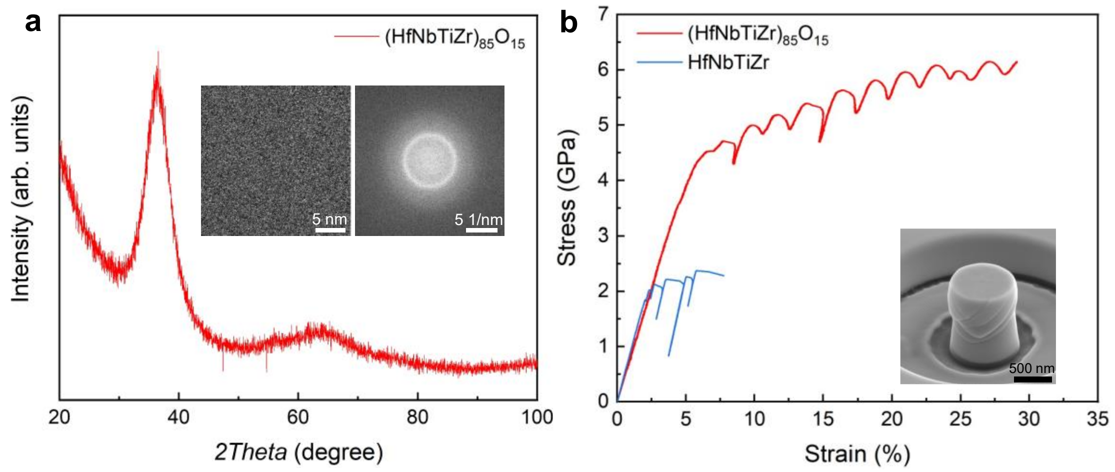

Supplementary Fig. 15| Microstructure and mechanical properties of amorphous $(HfNbTiZr)_{85}O_{15}$ MPEA. a XRD pattern and TEM image of the as-deposited film. **b** Typical compressive stress-strain curve and corresponding SEM image of deformed micropillar. The diameter of the as-fabricated micropillars is ~650 nm. The stress-strain curve of HfNbTiZr amorphous alloy[3] is also plotted for comparison. Source data are provided as a Source Data file.

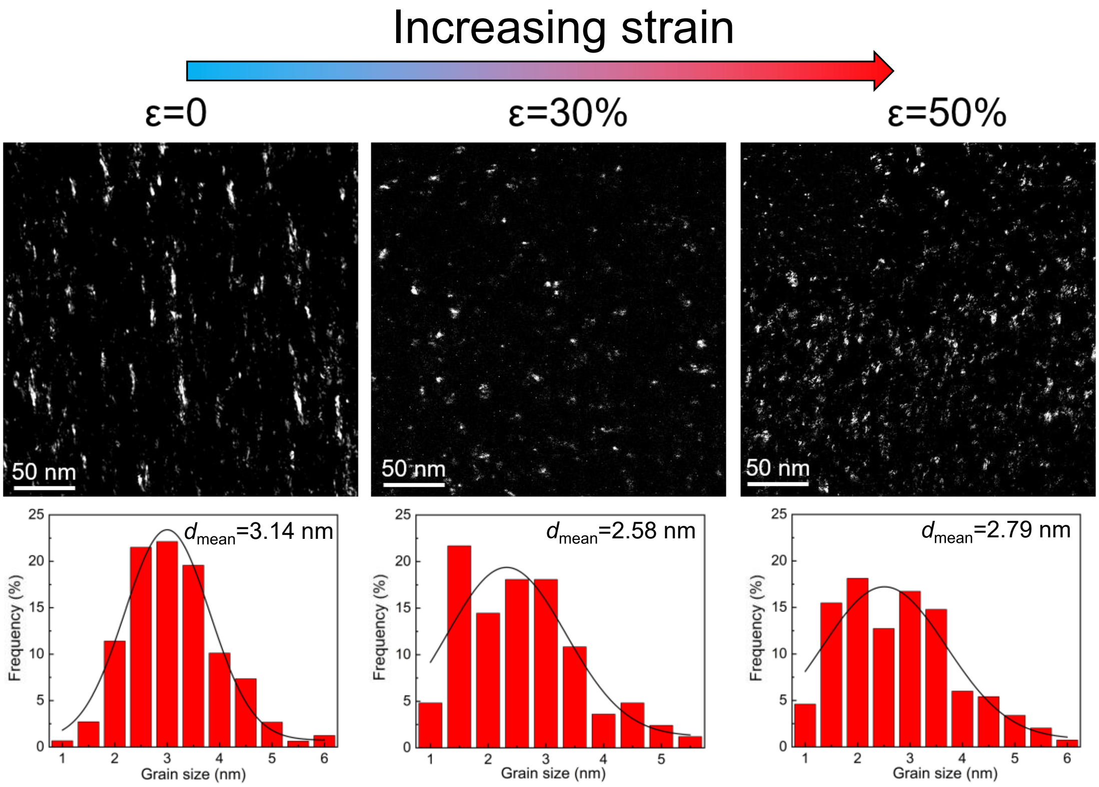

Supplementary Fig. 16| Evolution of grain morphology upon deformation. Typical DF-TEM images (upper panel) and corresponding grain size distributions (lower panel) of the compressed O-13 micropillars with increasing strain. Source data are provided as a Source Data file.

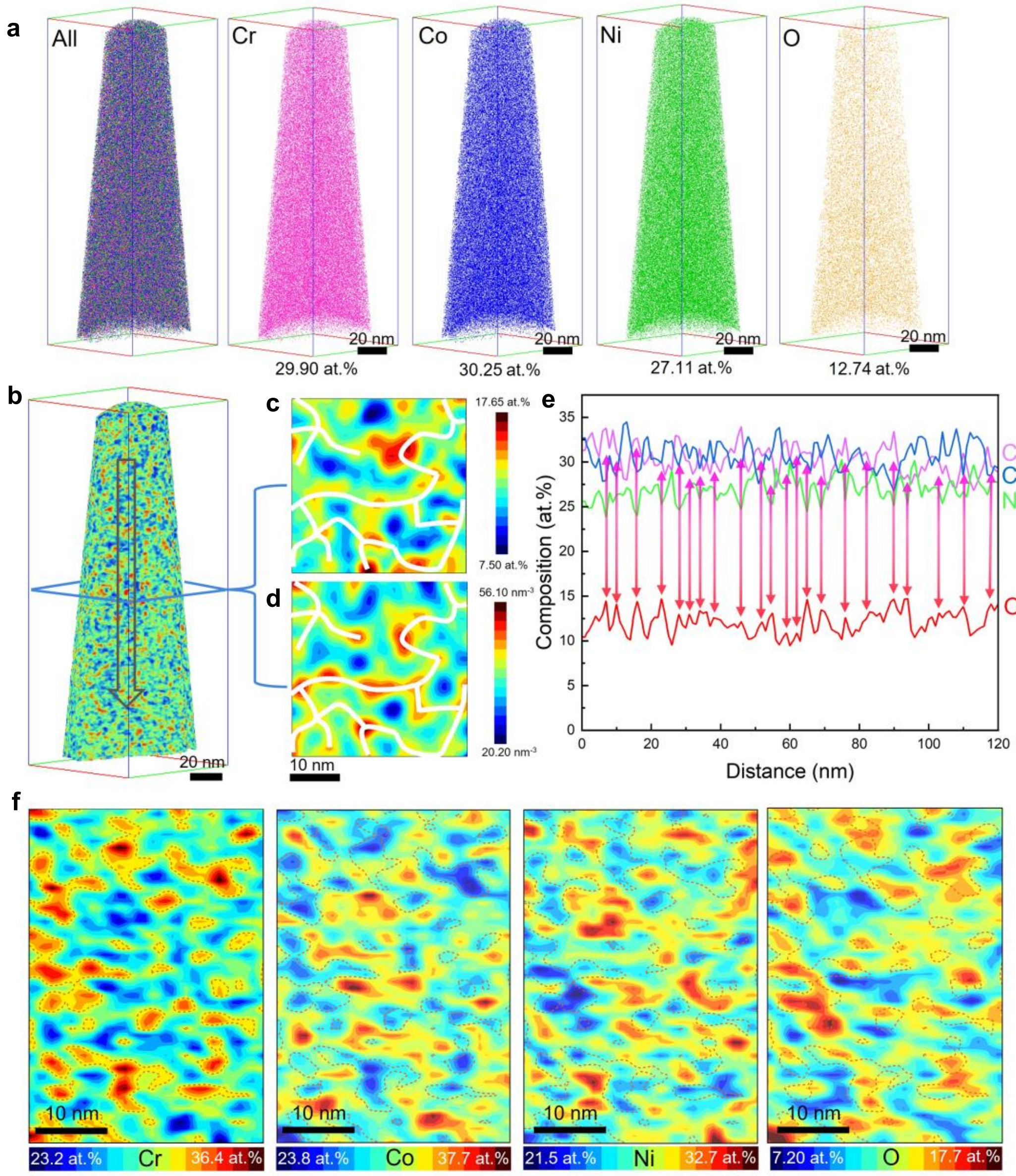

Supplementary Fig. 17| APT results of the O-13 micropillar after deformation. a Combined and individual 3D elemental map with chemical composition in atomic percent (at.%). **b** 3D reconstruction map from the APT measurements, illustrating the variation of the Cr concentration throughout the sample. **c-d** Two-dimensional in-plane concentration modulation (**c**) and atomic density (**d**) taken from the blue-framed cross-section in **b**. GBs are highlighted by white solid lines. **e** 1D compositional profile along the length direction of the arrows displayed in **b**. **f** 2D compositional plots of Cr, Co, Ni, and O in a 1 nm-thick slice from the APT dataset. The dotted orange lines on each plot correspond to the iso-concentration line of 30.50 at.% Cr. Source data are provided as a Source Data file.

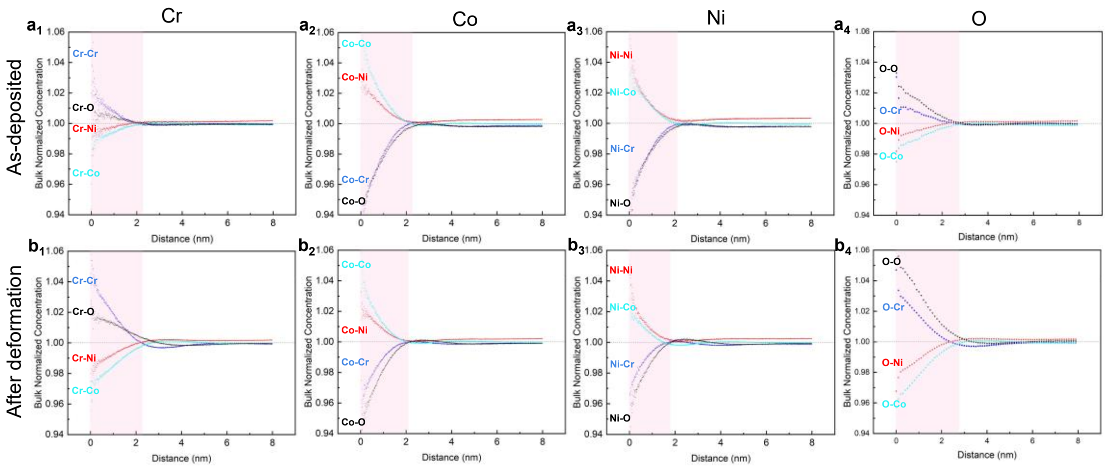

Supplementary Fig. 18| APT RDFs of the as-deposited and deformed O-13 samples. **a_1-a_4** and **b_1-b_4** are the calculated RDFs from the APT data with Cr, Co, Ni, and O as the center atoms, for as-deposited and deformed O-13 MPEAs, respectively. Note that the RDFs were normalized by the averaged composition for the APT-analyzed volume. Source data are provided as a Source Data file.

Supplementary Table 1| Values of electronegativity for O, Cr, Co and Ni elements according to the Pauling and the Allen scale[4], respectively.

Element	O	Cr	Co	Ni
Electronegativity (Pauling)	3.44	1.66	1.88	1.91
Difference with oxygen		1.78	1.56	1.53
Electronegativity (Allen)	3.61	1.65	1.84	1.88
Difference with oxygen		1.96	1.77	1.73

Supplementary Table 2| Summary of Hall-Petch fitting parameters. The tabulated parameters were obtained from linear regression, employing the conventional Hall-Petch relationship ($H = H_0 + kd^{-\frac{1}{2}}$) for the strengthening regime and a linear function ($H = H_0 + kd$) for the inverse Hall-Petch (softening) regime[5,6]. Here, H represents hardness, d is the grain size, and H_0 and k are fitting constants, reported as value ± standard error. The coefficient of determination (R^2) is used to indicate the goodness of fitting.

Line	Fitting Equation	H_0	k	R^2 (COD)
Hall-Petch line of CoCrNi	$H = H_0 + kd^{-\frac{1}{2}}$	2.39 ± 0.33	35.05 ± 1.51	0.97
Inverse Hall-Petch line of CoCrNi	$H = H_0 + kd$	7.06 ± 0.29	0.23 ± 0.04	0.80
Hall-Petch line of NiMo	$H = H_0 + kd^{-\frac{1}{2}}$	4.74 ± 0.20	4.20 ± 0.78	0.97
Inverse Hall-Petch line of NiMo	$H = H_0 + kd$	4.68 ± 0.10	0.13 ± 0.01	0.95
Hall-Petch line of NiCo	$H = H_0 + kd^{-\frac{1}{2}}$	3.21 ± 0.25	12.30 ± 1.98	0.93
Inverse Hall-Petch line of NiCo	$H = H_0 + kd$	3.86 ± 0.39	0.08 ± 0.02	0.94
Hall-Petch line of Ni	$H = H_0 + kd^{-\frac{1}{2}}$	2.00 ± 0.10	10.75 ± 0.24	0.99

Supplementary Table 3| Strain rate sensitivity (*m*) and activation volume (*V*) of the CoCrNi-O alloys.

CoCrNi-O alloys	Strain rate sensitivity (*m*)	Activation volume (m^3) $\times 10^{-29}$	Activation volume (b^3)
O-13	0.021	9.43	32
O-5	0.018	9.63	33
O-30	0.018	8.43	29

Supplementary Table 4| Sputtering parameters of the CoCrNi alloys with controlled grain sizes fabricated by magnetron sputtering.

CoCrNi alloys	1# 16.55	2# 10.94	3# 7.66	4# 6.86	5# 6.28	6# 4.30	7# 3.93	8# 3.86	9# 3.48	10# 2.09
Grain size (nm)	16.55	10.94	7.66	6.86	6.28	4.30	3.93	3.86	3.48	2.09
Sputtering pressure (Pa)	0.70	2.00	2.00	2.00	1.60	2.00	2.50	0.70	0.30	2.50
Sputtering power (W)	120	60	60	60	60	60	60	120	60	60
Distance between substrate and target (mm)	100	120	100	120	120	100	100	120	120	120
Substrate temperature (K)	298	498	408	298	298	298	298	298	298	298
Ar flow rate (sccm)	60	60	60	60	60	60	60	60	60	60

Supplementary Table 5| Sputtering parameters and grain sizes of CoCrNi-O alloys with different oxygen content.

CoCrNi-O alloys	O-13	O-5	O-30
Grain size (nm)	3.00	8.18	6.02
Oxygen content of sputtering target (wt.%)	0.31	0.02	0.02
Sputtering pressure (Pa)	0.30	0.30	0.30
Sputtering power (W)	60	60	60
Distance between substrate and target (mm)	120	120	120
Substrate temperature (K)	298	298	298
Ar flow rate/O_2 flow rate (sccm)	60/0	56/4	54/6

References

1. Andersson, J.-O., Helander, T., Höglund, L., Shi, P. & Sundman, B. Thermo-Calc & DICTRA, computational tools for materials science. *Calphad* **26**, 273-312 (2002).
2. Gammer, C., Mangler, C., Rentenberger, C. & Karnthaler, H. P. Quantitative local profile analysis of nanomaterials by electron diffraction. *Scr. Mater.* **63**, 312-315 (2010).
3. Wu, G. et al. Symbiotic crystal-glass alloys via dynamic chemical partitioning. *Materials Today* **51**, 6-14 (2021).
4. Allred, A. L. Electronegativity values from thermochemical data. *J. Inorg. Nucl. Chem.* **17**, 215-221 (1961).
5. Petch, N. J. The cleavage strength of polycrystals. *J. Iron Steel Inst.* **174**, 25-28 (1953).
6. Hall, E. The deformation and ageing of mild steel: III discussion of results. *Proc. Phys. Soc. B* **64**, 747 (1951).
